# Toward an Executive Origin for Acquired Phonological Dyslexia: A Case of Specific Deficit of Context-Sensitive Grapheme-to-Phoneme Conversion Rules

**DOI:** 10.3233/BEN-2012-129003

**Published:** 2013

**Authors:** Noémie Auclair-Ouellet, Marion Fossard, Marie-Catherine St-Pierre, Joël Macoir

**Affiliations:** ^1^Programme de Médecine ExpérimentaleFaculté de MédecineUniversité LavalSainte-FoyQCCanada; ^2^Centre de Recherche de I'Institut Universitaire en Santé Mentale de QuébecQuébecQCCanada; ^3^Institut des Sciences du Langage et de la CommunicationFaculté des Lettres et Sciences HumainesUniversité de NeuchâtelNeuchâtelSwitzerland; ^4^Programme de Maîtrise en OrthophonieDépartement de RéadaptationFaculté de MédecineUniversité LavalSainte-FoyQCCanada; ^5^Centre Interdisciplinaire de Recherche en Réadaptation et Intégration Sociale (CIRRIS)QuébecQCCanada

**Keywords:** Acquired dyslexia, phonological dyslexia, executive functions, grapheme-to-phoneme conversion rules

## Abstract

Phonological dyslexia is a written language disorder characterized by poor reading of nonwords when compared with relatively preserved ability in reading real words. In this study, we report the case of FG, a 74-year-old man with phonological dyslexia. The nature and origin of his reading impairment were assessed using tasks involving activation and explicit manipulation of phonological representations as well as reading of words and nonwords in which the nature and complexity of grapheme-to-phoneme conversion rules (GPC rules) were manipulated. FG also underwent an extensive neuropsychological assessment battery in which he showed impaired performance in tests exploring verbal working memory and executive functions. FG showed no phonological impairment, and his performance was also largely unimpaired for reading words, with no effect of concreteness, grammatical class, morphological complexity, length or nature and complexity of the GPC rules. However, he showed substantial difficulties when asked to read nonwords with contextual GPC rules. The contribution of FG’s executive deficits to his performance in reading is discussed.

## 1. Introduction

Phonological dyslexia is a written language disorder characterized by impairment in reading nonwords in the presence of a relatively preserved ability to read words [1]. Some theoretical proposals suggest that this impairment could be caused by a disruption of phonological processing [2], while others suggest that it could result from impairment of the nonlexical reading route, affecting grapheme-to-phoneme conversion (GPC) rules [3,4].

## 2. Methods

The case of FG, a 74-year-old right-handed man with eleven years of education is reported. He presented with acquired language deficits characterized by foreign accent syndrome, mild agrammatism, agraphia,

ISSN 0953-4180/13/$27.50 © 2013 – IOS Press and the authors. All rights reserved

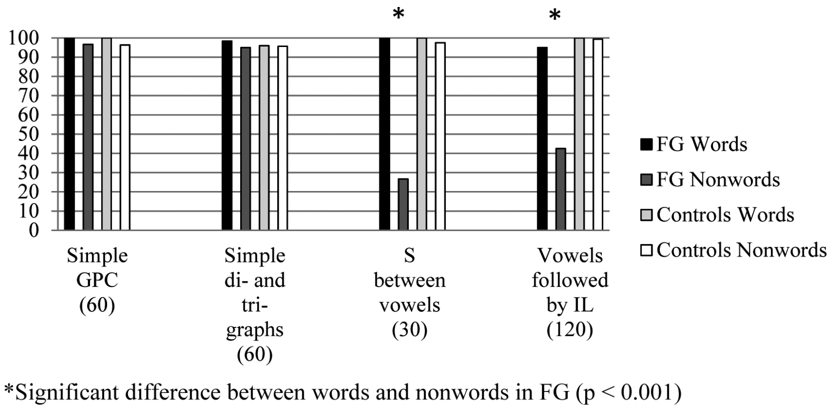

Fig. 1. Percentage of correct responses on tasks involving reading of words and nonwords according to the nature and complexity of GPC rules for FG and mean for controls. *Significant difference between words and nonwords in FG ($p < 0.001$).

and phonological dyslexia. FG completed a battery of neuropsychological tests including tasks involving attention, working memory, executive functions, visual-perceptual function, motor control and episodic memory.

The nature and origin of his reading impairment were assessed using tasks involving activation and explicit manipulation of phonological representations as well as reading of words and nonwords in which the nature and complexity of GPC rules were manipulated. The experimental list comprised stimuli with simple G-P correspondences in which graphemes have an unambiguous pronunciation (60 stimuli comprising letters with transparent GPC rules and 60 stimuli comprising di- and tri-graphs with consistent GPC rules), and stimuli with context-sensitive conversion rules in which the pronunciation does not derive from a simple grapheme-to-phoneme mapping but is directly dependent on the environment in which specific graphemes are presented. For these rules, the experimental list comprised 30 stimuli for the 'vowel + S + vowel rule' according to which the letter S, which is consistently pronounced /s/, changes to /z/ in an intervocalic position, and 120 stimuli for the "A, E, EU, OU + IL(L) rule" according to which the graphemes I + L(L), pronounced /i + l = il/ according to transparent GPC rules, are consistently pronounced /j/ when preceded by A, E, EU and OU in the middle or at the end of words, a complex GPC process that results in the following correspondences: A + IL(L) = /aj/; E + IL(L) = /ɛj/; EU + IL(L) = /œj/; OU + IL(L) = /uj/). FG's performance was compared to the results of five right-handed male controls matched for age and education level.

## 3. Results

FG's performance was normal on all the cognitive tasks except those evaluating working memory and executive functions, in which he performed below the mean of the control participants. He was unimpaired in activating and manipulating phonological representations. Figure 1 shows FG's performance in reading for different types of words and nonwords compared with the performance of the control participants. His performance was normal for words, regardless of the nature and complexity of the GPC rules. He also showed no difficulty with nonwords in which graphemes have an unambiguous pronunciation. However, his performance was substantially affected for nonwords with context-sensitive GPC rules. In these nonwords, FG produced errors resulting from difficulties in the application of the GPC rule that would have been appropriate in this context. For example, when asked to read a nonword like "lasate" (/lazat/), he pronounced it as /lasat/, which corresponds to the usual and consistent pronunciation of the grapheme S.

## 4. Discussion

FG presented with a profile of acquired phonological dyslexia never reported before. He showed no phonological impairment but was impaired when asked to read nonwords with contextual GPC rules. This pattern of performance led us to question the functional origin of FG's dyslexia

By resorting to the 'dual-route' model of reading [3, 4], FG's phonological dyslexia can be explained by the disruption of the nonlexical reading route affecting

GPC rules. However, it is also possible that the patient's concomitant executive deficits might have contributed to his performance in reading [5]. Executive functions refer to the ability to plan, initiate and control behaviors that are directed towards a goal, while having the flexibility to adjust this behavior when new information is presented [6]. Recent studies, mainly published in the field of developmental dyslexia, indicate that executive functions may have a specific involvement in reading [7,8].

In reading, FG showed impairments only with nonwords, namely, with contents for which he had no prior experience and that he could not process automatically. Moreover, his performance was characterized by a specific impairment in reading nonwords with context-sensitive GPC rules. Unlike words with consistent GPC rules in which letters are pronounced the same way regardless of the context, the pronunciation of words with context-sensitive GPC rules depends on the environment in which specific graphemes are presented. For example, the pronunciation of some graphemes requires taking into consideration the next letter, the two next letters, or the previous *and* the next letter. Furthermore, it is possible to conceive that, before the complex context-sensitive GPC rule is fully processed, different competing pronunciations are activated. Let us consider the non-word "lamail" (/lamaj/) in which the second syllable requires the application of a context-sensitive rule. The pronunciation that is generated by this rule could be viewed as being "updated" as the information coming from the context is integrated. Thus, reading the group M+A+I+L could activate the pronunciations for M+A (/ma/) and M+A+I (/me/ or /mɛ/) along the way, causing more interference. This explanation could account for the errors produced by FG (e.g., VANAIL pronounced /vanɛl/ instead of /vanaj/). In short, an executive explanation seems possible because FG showed impairment when he was confronted with unfamiliar material, and especially in contexts requiring him to maintain and manipulate a larger amount of information, and to inhibit competing responses.

Moreover, other aspects of FG's language performance tasks lend themselves to considering an executive explanation to account for his deficit in reading nonwords. In another study conducted with FG [9], an executive explanation was also considered to account for his inflectional morphology difficulties. While his performance was normal for real verbs, FG showed significant impairment when he was required to apply inflection rules to nonverbs and nonadjectives.

As a whole, our study underlines the need for studies about acquired phonological dyslexia in which the nature and specificity of GPC rules, as well as the executive and controlled processes, are considered.
